# Pre-Burn Health-Related Quality of Life: Patient and Partner Perspectives

**DOI:** 10.3390/ebj3010011

**Published:** 2022-02-11

**Authors:** Elise Boersma-van Dam, Helma W. C. Hofland, Alette E. E. de Jong, Nancy E. E. Van Loey

**Affiliations:** 1 Association of Dutch Burn Centres, 1941 AJ Beverwijk, The Netherlands; 2 Department of Clinical Psychology, Utrecht University, 3584 CS Utrecht, The Netherlands; loeyn2@maasstadziekenhuis.nl; 3 Association of Dutch Burn Centres, Maasstad Hospital, 3079 DZ Rotterdam, The Netherlands; hoflandh@maasstadziekenhuis.nl; 4 Red Cross Hospital, 1942 LE Beverwijk, The Netherlands; aeedejong@rkz.nl

**Keywords:** burns, health-related quality of life, agreement, retrospective study, proxy, prevalence adjusted kappa

## Abstract

A proxy-assessment of health-related quality of life (HRQL) may be an alternative for burn patients who are medically unable to self-report shortly after being admitted to the hospital. This study examined the patient–partner agreement on the recalled pre-injury HRQL of burn patients. In a multi-centre study of 117 patient–partner pairs, the recalled pre-burn HRQL was assessed with the EQ-5D-3L + Cognition during the acute phase following the burns. Agreement was evaluated with Kappa and ICC statistics. Burn severity and PTSD symptoms were assessed as potential predictors of disagreement. The results showed that pre-burn EQ-Index scores were similar to population norms, whereas the EQ Visual Analog Scale (EQ-VAS) scores of patients were significantly higher. Agreement varied across EQ-5D domains and, after adjusting for prevalence, was substantial to almost perfect. Average agreement on the EQ-Index and EQ-VAS was, respectively, substantial and moderate, but differences between partners were larger at lower levels of HRQL, and specifically in the pain/discomfort domain. Patient–partner differences could not be explained by the patient’s age or gender, number of surgeries, partner’s presence at the burn event, or post-traumatic stress disorder (PTSD) symptoms of either the patient or partner. In conclusion, patient–partner agreement is substantial and partner–proxy reports of pre-burn EQ-5D domains and EQ-Index scores may be used to complement or serve as a substitute for the patient’s assessment. Given the moderate agreement on the EQ-VAS, it may be less suited for proxy assessment.

## 1. Introduction

A severe burn injury can vastly impact the patient’s physical, psychological, and social well-being. Consequently, reduced levels of health-related quality of life (HRQL) [1] are common within the first year post-burn [2]. A growing number of studies have assessed pre-burn HRQL [3,4], which may serve as a reliable benchmark for patient recovery of HRQL and may aid prognosis and treatment choices [5]. Since sustaining burns is an unexpected event, pre-burn functioning is preferably assessed retrospectively and within the first weeks after the injury [6,7]. Compared to a prospective assessment, a retrospective assessment carries the risk of recall bias, but prevents bias related to scale recalibration or response shift [8]. To estimate pre-burn health status more accurately, partner–proxy assessment may complement the patient’s self-reporting, since partners are well aware of the patient’s pre-burn functioning. Alternatively, the partner’s proxy assessment may serve as a substitute for the patient’s assessment if medical status prevents timely self-report by the patient. In that case, data collection by proxy may avoid systematic non-participation and reduce missing data [7,9]. 

In general, proxies of critically ill patients tend to report lower levels of HRQL than patients, both prospectively [10,11] and retrospectively [12,13]. Furthermore, patients usually report pre-injury HRQL levels that are higher than population norms [14]. Proxy agreement of (pre-injury) HRQL has not been studied in the adult burn population, but studies in intensive care unit (ICU) patients have shown varying levels of pre-injury agreement, ranging from slight-to-moderate [13,15,16] to moderate-to-high agreement (e.g., [17,18]). 

For clinical decision making, reliance on proxy judgements in the absence of self-report may have significant implications for the evaluation of the success of care and treatment. To better understand the origin of proxy–patient disagreement and to estimate whether the partner is a reliable proxy, it is important to study factors that may predict disagreement [9,19]. First, the level of HRQL impairment may impact agreement, in either a linear or u-shaped relation [9]. Some empirical research shows lower levels of agreement in relation to more impaired functioning [9,19,20], while another study showed larger discrepancies for patients with moderately impaired health rather than for those with either good or poor health [21]. Second, it is important to study the relation between burn severity and patient–partner disagreement to establish the accuracy of proxy reports for more severely burned patients. Indeed, proxy–patient agreement may be most relevant for patients who are medically unable to self-report their HRQL [9]. Third, agreement may partly depend on the concreteness of the domains under consideration. That is, proxies may have most difficulty in assessing the more subjective domains (e.g., anxiety and depression) compared to more objective physical aspects (e.g., mobility and self-care [17,20]). Fourth, increased levels of post-traumatic stress disorder (PTSD) symptoms may be related to patient–partner differences. Increased levels of PTSD symptoms have been reported in both patients with burns [22] and their partners [23,24], and memory disturbances, which are part of the PTSD diagnostic criteria [25], have been related to its symptoms [26]. Empirical evidence shows that increased PTSD symptom levels were related to changes in recalled pre-injury HRQL over a period of 12 months [27], exemplifying the possible effect of PTSD symptoms on the recollection of pre-injury HRQL. 

The aim of the current study was twofold. First, to compare recalled pre-burn HRQL from patients and their proxies (partners) to population norms and second, to evaluate patient–partner agreement on the recall of a patient’s pre-burn HRQL and study factors related to discrepancies. In line with relevant factors reported in the literature, the effect of HRQL impairment, burn severity, and PTSD symptoms on patient–partner differences was studied.

## 2. Materials and Methods

### 2.1. Inclusion and Procedure

The data in this study were collected as part of a larger project concerning the social impact of burns. All consecutively admitted patients and their partners were invited to participate by a local researcher during the patient’s stay in one of the three Dutch or three Belgian burn centres. Recruitment took place between October 2013 and October 2015. The patients’ pre-burn HRQL data were also part of previous work that described patients’ recovery to pre-burn HRQL [28]. In the current study, a subsample of patients with a participating partner was studied. The larger project was approved by the ethics boards in The Netherlands and Belgium (NL44682.094.13 and B670201420373). Inclusion criteria for patients were a hospital stay of >24 h following the burn event, age ≥18 years and proficiency in Dutch. The latter two criteria also applied to the partners. Exclusion criteria were psychiatric problems that interfere with the comprehension of questionnaires (e.g., psychosis, cognitive problems), and inhalation injury without external burns. After receiving oral and written study information, the participants provided informed written consent. 

### 2.2. Measures

#### 2.2.1. Recalled Pre-Burn Health-Related Quality of Life

The EQ-5D-3L + cognition [29] is a self-report scale used to assess generic HRQL and was administered in the acute phase following the burn injury (M_patients_ = 22 days post-burn, SD = 23; M_partners_ = 24 days post-burn, SD = 24). The HRQL was assessed along five single-item health domains: mobility, self-care, usual activities, pain and anxiety/depression as well as the added cognition domain, which measured the extent to which the patient experienced problems with memory and concentration. For each domain, patients and partners independently recalled the patient’s health before the burn event from their own perspective. Answers were rated on a 3-point scale: “no problems”, “moderate problems”, or “severe problems”. The first five domains were combined into the EQ-5D Index (EQ-Index) using calculations based on the European Visual Analog Scale (VAS) value set [30]. The resulting EQ-Index ranges from 0 “death” to 1 “full health”. In addition, the EQ-5D includes an EQ-VAS that is scaled vertically and runs from 0 (worst imaginable health state) to 100 (best imaginable health state). The EQ-5D, which is short and easy to complete, is often used after burns [31] and has good feasibility and reasonable criterion validity in the burn population [32]. The addition of a cognition domain slightly improved the psychometric performance of the EQ-5D in traumatic brain injury patients [33].

#### 2.2.2. Posttraumatic Stress Disorder Symptoms

The Impact of Event Scale-Revised (IES-R; [34]) was used to assess patient and partner PTSD symptoms in the acute phase following the burn event. The IES-R is a 22-item self-reporting questionnaire that measures three PTSD symptom clusters––intrusion, avoidance and hyper-arousal––over the previous week. Answers were given on a 5-point Likert scale and summed to obtain a total score, and sumscores ≥ 33 indicated a possible diagnosis of PTSD [35]. If at least 19 of the 22 items were completed, the sum scores were calculated based on the mean of the completed items. The IES-R was validated in Dutch trauma populations and showed good psychometric properties [36]. Reliability of the IES-R in the current study was excellent, with a Cronbach’s alpha of 0.95 for patients and 0.93 for partners. 

#### 2.2.3. Burn Characteristics

The total body surface area (TBSA) burned, number of surgeries and need for mechanical ventilation were recorded from the medical file. The TBSA is the estimated percentage of the body covered with partial and full thickness burns. The number of surgeries indicates the number of skin graft procedures that was required to cover the wounds, and it is used as an indicator of burn severity. Presence at the burn event was self-reported by the partner.

### 2.3. Statistical Analysis

First, the pre-burn EQ-Index and EQ-VAS scores of patients and their partners were compared to gender-and-age adjusted population norms from a national representative sample of the non-institutionalized adult population of their country: Belgium or The Netherlands [37]. One-sample Student’s *t*-tests were used to test the differences for significance.

Second, agreement between patients and partners on the six domains of the EQ-5D was assessed using Cohen’s Kappa with 95% confidence intervals. Linear weights were applied to account for the ordinal structure in the data [38], and a prevalence-adjusted weighted kappa [39] was reported. A prevalence effect may be present if the majority of the sample reported the same response option, (e.g., “no problems”) on a given EQ-5D domain, causing large absolute differences among the counts in the cells of agreement (i.e., the cells on the diagonal of a cross-table). These differences increase the proportion of agreement expected by chance, thereby reducing the kappa, even with a large proportion of absolute agreement. To obtain a prevalence-adjusted kappa (PAK) the cells of agreement were replaced by their combined average before calculating the weighted kappa [39]. For the EQ-Index and EQ-VAS, agreement was assessed by the intraclass correlation coefficient (ICC) using a two-way random effects model for absolute agreement [40]. Differences in the EQ-Index and EQ-VAS were also compared to the minimally important difference (MID), which indicates the minimum change that reflects a clinically relevant change in the HRQL. For the EQ-Index, a MID of 0.074 was used, based on a study in patients with a wide range of medical conditions [41]. For the EQ-VAS, an MID of 8 was chosen, based on several studies in specific (non-burn) patient populations [42,43,44]. The MIDs were established using both anchor- and distribution-based methods. For the kappa and ICC coefficients, 0.00 to 0.20 was considered slight, 0.21 to 0.40 fair, 0.41 to 0.60 moderate, 0.61 to 0.80 substantial, and 0.81 to 1.00 almost perfect agreement [45]. 

Third, differences between patients and partners regarding EQ-Index and EQ-VAS scores were further tested using paired Student’s *t*-tests. Bland–Altman plots were generated to visually inspect the difference between patients and partners in relation to the combined mean of the patient and partner responses [46]. The mean difference and limits of agreement (95% confidence interval) were also calculated. Furthermore, the effects of gender, age, burn severity, partner’s presence at the burn event, and PTSD symptoms on patient–partner differences on the EQ-Index and the EQ-VAS were examined in multiple regression analyses. A *p*-value < 0.05 was considered significant. Analyses were performed using IBM SPSS 24 (IBM Corp., Armonk, NY, USA).

## 3. Results

### 3.1. Descriptive Analyses

The final sample consisted of 117 pairs of patients with burns and their partners. Of the 266 originally participating patients, 71 reported no partner and 8 did not say. Of the remaining 187 patients with a partner, 117 (62.6%) couples completed the pre-burn HRQL measure and were included in the study. Comparing the 117 included couples to the 70 couples with incomplete data and to the 79 patients without a partner, no statistically significant differences (*p* > 0.05) were observed with respect to patient’s gender, patient’s reported pre-burn and post-burn HRQL, TBSA burned, and number of surgeries. However, the 79 patients without a partner were significantly younger (M = 39.2, SD = 16.9) than the included patients (M = 45.8, SD = 15.1, *p* = 0.012) and the patients with incomplete couple data (M = 45.7, SD = 14.5, *p* = 0.035).

The patients had a mean age of 45.8 years (SD = 15.1); for partners this was 44.4 years (SD = 14.4). Most pairs consisted of a male patient and a female partner (*n* = 90, 76.9%); the remainder were female patients with a male partner (*n* = 27, 23.1%). The patient’s mean total body surface area (TBSA) burned was 10.4% (SD = 11.1, range 1.0–75.0). The median number of surgeries was 1 (range 0–14). For further analyses, this variable was categorised as: “no surgeries” (*n* = 53; 45.3%), “one surgery” (*n* = 39; 33.3%) or “more than one surgery” (*n* = 25; 21.4%). Forty-four partners (40%) were present at the burn event. The number of patients with clinically relevant levels of PTSD symptoms was 21 (18.1%); for partners, this was 34 (29.1%). One patient did not complete the IES-R. 

### 3.2. Recalled Pre-Burn HRQL Compared to Population Norms

Table 1 describes the average pre-burn EQ-Index and EQ-VAS, reported by both patients and their partners. Compared to age, gender and country specific population norms [37], the EQ-Index from both patients and partners did not deviate significantly from the adjusted norm. Patients’ EQ-VAS was on average 4 points higher than the adjusted population norms, which was a significant difference but within the bounds of the MID. Partners’ EQ-VAS did not differ significantly from the population norms.

### 3.3. Agreement

Table 2 shows that most patients and partners reported no problems in a specific EQ-5D domain. Exact agreement was around 90% for most domains except for pain (71.8%). Agreement based on the weighted kappa, ranged from slight to substantial for the six EQ-5D domains. After adjustment for prevalence, the weighted kappa (PAKw) was considerably higher and indicated substantial-to-almost-perfect agreement for the six domains. Agreement according to the PAKw was the lowest for pain/discomfort and highest for mobility. Exact agreement for the EQ-Index was acceptable, but exact agreement for the EQ-VAS was low even if defined within the borders of the MID. The single-rater ICC for the EQ-Index and EQ-VAS was, respectively, moderate and fair, and the average inter-rater ICC was substantial and moderate.

### 3.4. Predictors of Differences on the EQ-Index and the EQ-VAS

**Figure 1:**
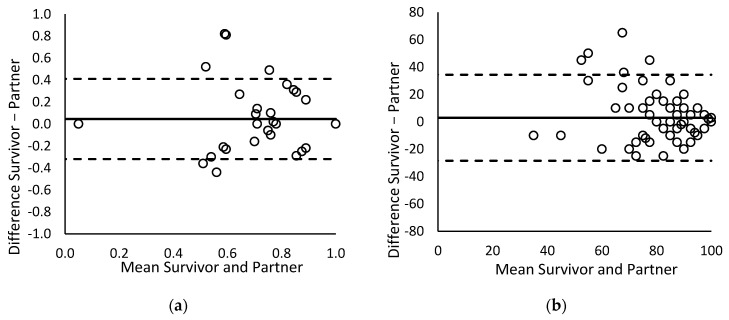
Bland–Altman plots of differences between patient and partner ratings against the mean of both ratings for (**a**) the EQ-Index and (**b**) the EQ-VAS. The horizontal lines represent the mean difference and limits of agreement (95% Confidence Interval around the mean difference).

Patients reported significantly higher pre-burn EQ-Index scores than their partners, (*t*(116) = 2.93, *p* = 0.009), but the mean paired difference of 0.05 (see Table 1) lies within the MID. The mean paired difference on the EQ-VAS was 3.0 and was not significant (*t*(106) = 1.89, *p* = 0.062). To examine the relation between patient–partner differences and possible explaining factors, differences on the EQ-Index and EQ-VAS were regressed on the couple’s mean score, patient’s age and gender, number of surgeries, partner’s presence at the burn event, and PTSD symptoms of both patients and partners. The regression models were not significant for either the EQ-Index, (*F* (8, 100) = 0.83, *p* = 0.58, *R*^2^ = 0.25) or the EQ-VAS, (*F* (8, 90) = 1.50, *p* = 0.17, *R*^2^ = 0.34), indicating that the predictors did not significantly add to the prediction of systematic differences. *Absolute* differences between patients and partners were significantly correlated to couple’s mean scores for both the EQ-Index (*r* = −0.61, *p* < 0.001) and EQ-VAS (*r* = −0.53, *p* < 0.001), indicating that differences between patients and partners tended to be larger at lower EQ-Index and EQ-VAS scores. The Bland–Altman plots of actual differences between patients and partners in Figure 1 illustrate this relationship. The plots also show that on both the EQ-Index and the EQ-VAS, extreme differences were reported that fell outside the confidence intervals. 

## 4. Discussion

This study examined patient–partner agreement on the retrospectively recalled pre-burn HRQL. EQ-Index scores from both patients and partners were comparable to population norms, whereas the patients’ EQ-VAS scores were higher. Agreement varied across the six EQ-5D domains, but after adjustment for prevalence, it was substantial to almost perfect. The average inter-rater agreement on the EQ-Index and EQ-VAS was, respectively, substantial and moderate. On average, patients reported a higher pre-burn EQ-Index compared to partners. Differences were larger at lower HRQL levels, but could not be explained by demographic or burn-related factors or by the PTSD symptoms of patients and partners. 

Compared to age- and gender- adjusted population norms, the EQ-Index scores from both perspectives were comparable to the norm scores, but patients’ EQ-VAS scores exceeded the norms, whereas the partners’ did not. However, post hoc analyses excluding three extremely low-scoring partners (with high-scoring patients), showed that, on average partners may also exceed population norms. This was in line with studies that reported a pre-injury HRQL above population norms [14,47,48]. Deviation from the norms on the EQ-VAS, but not the EQ-Index, may be related to a more subjective interpretation of the EQ-VAS compared to the more objectively rated EQ domains [8], which makes the EQ-VAS more prone to recall bias and response shift. Recall bias may cause inflated pre-burn EQ-VAS scores, because of the idealization of the pre-burn situation [14]. Alternatively, and especially in patients themselves, the experience of the burn event may cause a response shift—an change in the internal standards of what constitutes “good” health [49,50]—resulting in a more highly recalled EQ-VAS than in the (mostly uninjured) norm population, and possibly making comparisons to the norm populations less valid. 

The EQ-Index outperformed the EQ-VAS on both the single-rater and the average inter-rater agreement. Single-rater agreement on the EQ-Index and EQ-VAS was, respectively, moderate and fair, and the average inter-rater agreement was substantial and moderate. However, the ICC estimates may have been compromised by three extremely low scoring partners. Indeed, post hoc analyses, excluding these cases, showed that all ICC estimates increased by about 0.10. Agreement according to the unadjusted weighted kappa was only fair in most individual domains. These results fit within the wide range of agreement levels reported in the ICU literature (e.g., [13,15,16,17,18]), and are the first assessment of proxy agreement in the adult burn population. However, the current study showed that the kappa for pre-burn HRQL is highly affected by prevalence [39] since the vast majority of patients and partners reported no problems in any EQ-5D domain. Adjustment for prevalence revealed almost perfect agreement between patients and partners on mobility, self-care, usual activities, and anxiety/depression, and substantial agreement for pain/discomfort and cognition. Agreement was the lowest on the pain/discomfort domain, which is not surprising, given that pain is a subjective experience that cannot directly be observed by others [51]. Besides, the experience of severe burn-related pain may have especially recalibrated the patient’s interpretation of pain [8,49], while the partner’s interpretation may not have changed that much, resulting in disagreement on pre-burn levels of pain. The results partly support previous findings, showing that proxies have more difficulty in assessing subjective domains compared to more the objective physical aspects of HRQL [17,20] although (prevalence adjusted) agreement on anxiety/depression was high in the current study. 

The analyses of patient–partner differences showed that patients reported higher levels of pre-burn HRQL than partners, in line with the ICU literature [12,13]. However, post hoc inspection of the data revealed that these differences were partly driven by a few partners who reported an extremely low pre-burn HRQL. These partners may have erroneously reported on the post-burn HRQL of the patient. Nevertheless, the Bland–Altman plots showed that differences between the two informants were larger at lower levels of HRQL. Although this supports the idea of a linear relationship between agreement and functioning, we cannot exclude the existence of a u-shaped relationship due to the lack of data in the lower HRQL spectrum [9]. Furthermore, it should be noted that the relationship between absolute differences and a couple’s mean HRQL scores may also be the consequence of a ceiling effect because the majority of couples reported a perfect pre-burn health state (i.e., the maximum of 1 on the EQ-Index), preventing variability at the higher end of the spectrum. Differences on the EQ-Index and EQ-VAS were not related to burn severity, indicating that proxy reports were similarly accurate for severely and less severely burned patients. Furthermore, differences were also not explained by the PTSD symptoms of patients or partners, indicating that the symptoms did not disturb agreement in any direction. Further research should investigate patient–partner agreement and differences with respect to the post-burn HRQL because it may well be that these factors do relate to post-burn differences. 

Some limitations of this study should be noted. First, the sample was mostly restricted to patients with good pre-burn health, limiting generalizability to those who have low levels, especially since the results showed that differences between patients and partners increased at lower levels of a pre-burn HRQL. Second, the results cannot be generalized over other close relatives because the sample was limited to partners. Future research could compare multiple proxy perspectives (e.g., adult children, parents and siblings) to evaluate the eligibility of each of these proxies. Third, the partners were only asked to estimate a patient’s pre-burn health status, which precludes conclusions about agreement on post-burn HRQL. An assessment of both the pre-burn and post-burn HRQL by the partners could also have reduced the possibility of their erroneously evaluating the current HRQL instead of the pre-burn HRQL, which could explain some of the extreme differences between patients and partners in the current study. Fourth, about half of the patients and partners completed the pre-burn assessment after the recommended two weeks post-injury [7], which may have increased the possibility of recall bias. Although we cannot rule out recall bias on the EQ-5D Index, the average recalled pre-burn scores of both patients and partners did not differ from prospectively assessed population norms, which is a preliminary indication that no substantial recall bias (or response shift) occurred for the EQ-Index. Recall bias and response shift more likely have occurred on the EQ-VAS because of the subjective nature of this scale. Future research could investigate the effects of recall bias and response shift on the retrospective assessment of pre-burn functioning.

In conclusion, this study demonstrated that patient–partner agreement on the pre-burn HRQL is substantial. These results are a first indication that partner reports of pre-burn EQ domains may be reliably used to complement or, if needed, substitute for the patient’s assessment for research purposes. For monitoring the patient’s recovery in clinical practice, partner assessment may also substitute for the patient’s assessment if the patient is medically not able to self-report. However, the pain/discomfort domain may be more difficult to assess for partners, so it should be interpreted with caution. Furthermore, the EQ-VAS may be less suited for proxy reporting because of its subjective interpretation and lower level of agreement. Further research on proxy agreement is needed to evaluate the use of a proxy in the post-burn context and to evaluate the eligibility of other close relatives as proxies.

## Figures and Tables

**Table 1:** Descriptive and one-sample *t*-test results for the comparison of pre-burn EQ-Index and EQ-VAS of patients and partners (proxy) with population norms.

Description	*N*	*M*	*SD*	Population Norm ^1^	*t*	*df*	*p*
EQ-Index							
Patients	117	0.93	0.17	0.90	1.73	116	0.087
Partners	117	0.88	0.19	0.90	−1.12	116	0.26
EQ-VAS							
Patients	113	85.79	12.98	81.56	3.56	112	0.001
Partners	110	83.05	16.01	81.55	0.98	109	0.33

^1^ Age- and gender- adjusted population norms [37].

**Table 2:** Agreement between patients and partners on pre-burn EQ domains, EQ-Index and EQ-VAS.

Patient Response ^1^	Partner Response ^1^			Exact Agreement	K_w_	95% CI K_w_	PAK_w_	95% CI PAK_w_
	No	Some	Extreme					
Mobility				93%	0.64	0.39–0.88	0.92	0.87–0.98
No	102	5	0					
Some	3	6	0					
Extreme	0	0	1					
Self-care				92%	0.28	−0.17–0.73	0.91	0.86–0.97
No	107	7	0					
Some	2	0	0					
Extreme	0	0	1					
Usual Activities				89%	0.36	0.07–0.64	0.85	0.77–0.93
No	101	7	2					
Some	3	2	0					
Extreme	0	1	1					
Pain/Discomfort				72%	0.32	0.15–0.49	0.66	0.56–0.77
No	75	22	1					
Some	6	8	0					
Extreme	0	4	1					
Anxiety/ Depression				90%	0.34	0.08–0.60	0.87	0.80–0.94
No	102	7	1					
Some	3	3	1					
Extreme	0	0	0					
Cognition				89%	0.18	−0.11–0.46	0.78	0.66–0.89
No	102	7	0					
Some	6	2	0					
Extreme	0	0	0					
			**Agreement** ≤ **MID**	**Exact Agreement**	**ICC** **_single- rater_**	**95% CI**	**ICC** **_average rater_**	**95% CI**
EQ-Index			64%	62%	0.45	0.29–0.58	0.62	0.45–0.73
EQ-VAS (*n* = 107)			43%	22%	0.39	0.22–0.54	0.56	0.36–0.70

^1^ The exact wording of the response options of the EQ-5D varied over the domains, but for reasons of clarity and uniformity, responses have been labelled “no (problems)”, “some (problems)”, and “extreme (problems)”; PAK = prevalence-adjusted kappa; CI = confidence interval; MID = minimally important difference; ICC = intraclass correlation coefficient.

## Data Availability

The data presented in this study are available on request from the corresponding author. The data are not publicly available due to them containing information that could compromise participant privacy.
